# Improving the Nutritional Quality of Protein and Microbiota Effects in Additive- and Allergen-Free Cooked Meat Products

**DOI:** 10.3390/foods13121792

**Published:** 2024-06-07

**Authors:** Pablo Ayuso, Jhazmin Quizhpe, Fani Yepes, Domingo Miranzo, Antonio Avellaneda, Gema Nieto, Gaspar Ros

**Affiliations:** 1 Department of Food Technology, Nutrition and Food Science, Veterinary Faculty, University of Murcia, Regional Campus of International Excellence “Campus Mare Nostrum”, Espinardo, 30100 Murcia, Spain; pablo.ayuson@um.es (P.A.); jhazminedith.quizhper@um.es (J.Q.); gnieto@um.es (G.N.); 2 Cátedra de Seguridad y Sostenibilidad Alimentaria Grupo Fuertes, Universidad de Murcia, 30003 Murcia, Spainantonio.avellanedagoicuria@elpozo.com (A.A.)

**Keywords:** additives, cooked turkey breast, cooked ham, protein quality, in vitro digestibility, gut microbiota, short-chain fatty acids

## Abstract

The primary objective of the meat industry is to enhance the quality and positive attributes of meat products, driven by an increasing consumer demand for healthier, less processed options. One common approach to achieving this goal is the replacement of additives and allergens with natural ingredients. Nevertheless, the nutritional impact of these changes has not been extensively studied. To address these gaps, two new meat products were developed: cooked turkey breast and cooked ham. The products in question exclude additives and allergens and instead incorporate a blend of natural extracts containing vitamin C, chlorogenic acids, hydroxytyrosol, catechins, epicatechins, vinegar, and inulin fibre. The objective of this study was to evaluate the impact of these reformulations on protein quality and gut microbiota. Protein quality was evaluated using the Digestible Indispensable Amino Acid Score (DIAAS) following in vitro digestion. The microbial composition and short-chain fatty acid (SCFA) production were analysed through in vitro colonic fermentations in both normal-weight and obese participants in order to gauge their effect on gut microbiota. The results demonstrated that the reformulation of cooked turkey breast increased its digestibility by 6.4%, while that of cooked ham exhibited a significant 17.9% improvement. Furthermore, protein quality was found to have improved significantly, by 19.5% for cooked turkey breast and 32.9% for cooked ham. Notwithstanding these alterations in protein digestibility, the microbial composition at the phylum and genus levels remained largely unaltered. Nevertheless, total SCFA production was observed to increase in both groups, with a more pronounced effect observed in the normal-weight group. In conclusion, the substitution of artificial additives with natural ingredients in reformulated cooked meat products has resulted in enhanced digestibility, improved protein quality, and increased production of short-chain fatty acids.

## 1. Introduction

The consumption of meat, particularly red and processed meat, is increasingly discouraged by numerous scientists and global organisations [1]. This negative perception is primarily driven by ethical and cultural concerns associated with meat consumption, as well as the perceived link between high meat intake and increased risks of obesity and non-communicable diseases [2]. Nevertheless, meat continues to be an indispensable component of human evolutionary diets, offering essential high-quality protein and micronutrients [3]. Its high content of polyunsaturated fatty acids, minerals, and vitamins such as B6 and B12 [4,5] highlights its significance in human nutrition.

Due to the perishable nature of processed meat, various additives are commonly used for sensory appeal and preservation purposes. Additives are incorporated into food products for the purpose of maintaining their technological properties, preventing microbial contamination, adjusting acidity, and serving as thickeners, emulsifiers, and stabilisers [6,7]. The term “processed meat” encompasses a diverse range of products, the composition of which undergoes modifications through various processes that alter their properties [6]. Antioxidants, binders, emulsifiers, antimicrobials, stabilisers, flavourings, and colourings are among the most prevalent additives in meat products [8]. The European Food Safety Authority (EFSA) exercises rigorous control over the use of these additives, ensuring their safety and authorising their use in specified quantities for meat production.

There is a continuing debate regarding the potential health implications of additives used in meat products, given the lack of long-term studies in this area. It is noteworthy that antioxidants such as sodium citrate (E-331) and sodium erythorbate (E-316), in addition to acidity regulators including triphosphates (E-451), have demonstrated no adverse effects or significant toxicity in healthy individuals [9,10]. However, controversy surrounds carrageenans (E-407). While some studies suggest that they promote beneficial bacterial genera for gut health [11], others link them to intestinal inflammation and inflammatory bowel diseases in animal models [12,13]. Another contentious additive is sodium nitrite (E-250). While it may regulate arterial hypertension through the nitric oxide pathway [14], its use in meat products has raised concerns due to its potential to form N-nitrosamines, compounds known to be carcinogenic [15].

Recent findings have heightened consumer distrust in products containing additives, often perceived as less ‘natural’ [16]. With rising consumer interest in less processed, additive-free, and allergen-free options, the meat industry faces a critical challenge in finding natural alternatives. Natural extracts have emerged as successful replacements for traditional additives due to the negative reputation of synthetic counterparts [17,18]. Research has demonstrated that natural extracts rich in polyphenols like chlorogenic acid, catechins, epicatechins, or hydroxytyrosol [19,20,21] can effectively substitute certain antioxidant additives, enhancing nutritional properties and providing health benefits to consumers [22,23].

Despite the growing demand for natural products, it is imperative that any reformulation does not compromise the safety, technological, nutritional, and health attributes of meat products. Meat and meat products stand out as superior sources of high-quality protein compared to most vegetable and other animal sources [24]. Since 2013, the Food and Agriculture Organization (FAO) has employed the Digestible Indispensable Amino Acid Score (DIAAS) [25] to assess protein quality. This index introduces ‘protein digestibility’, reflecting the percentage of absorbed amino acids up to the terminal ileum. Currently, DIAAS values serve as the benchmark for meeting amino acid requirements per gram of protein. Most meat products boast DIAAS values exceeding 100, affirming their excellence as protein sources [26]. Regarding meat nutritional quality and products, pork is a rich source of high-quality protein and select nutrients [27], and turkey (specially breast muscles) has also revealed a high protein content (about 25%) and a high proportion of essential amino acids [28]. However, it remains crucial to ascertain whether replacing additives and allergens affects the DIAAS values of meat products.

Eliminating additives and allergens from meat products using natural extracts could profoundly impact gastrointestinal health by altering the gut microbiota. The term microbiota refers to the diverse community of bacteria, archaea, and eukaryotic microorganisms that inhabit the gastrointestinal tract. Over millennia, these species have co-evolved with their hosts, establishing a complex and mutually beneficial relationship [29]. The composition of gut microbiota is influenced by various factors such as diet, genetics, weight, stress, and medication [30], and it plays a crucial role in human physiology through microbial metabolism and interactions with the intestinal mucosa [31].

The dominant phyla in the gut microbiota, Firmicutes and Bacteroidetes, are involved in carbohydrate metabolism and the production of short-chain fatty acids (SCFAs) [32], which possess anti-inflammatory and immunoregulatory properties [33]. Changes in additives and allergens in meat products can impact the composition of gut microbiota [34,35].

Given the growing public concern over additives in meat products, it is essential to analyse how replacing these additives with natural ingredients affects the nutritional properties of meat. The hypothesis of this study posits that eliminating E-number additives and allergens from meat products and substituting them with a blend of natural herbal extracts containing vitamin C, chlorogenic acids, hydroxytyrosol, catechins, epicatechins, vinegar, and inulin fibre will enhance their nutritional value and positively influence gut microbiota. To validate this hypothesis, the reformulated products underwent rigorous nutritional analysis. Digestibility and protein quality were assessed through in vitro digestions, followed by examinations of changes in microbial composition and SCFA production via in vitro fermentations.

## 2. Materials and Methods

### 2.1. Reagents

The following chemicals were purchased from Sigma-Aldrich (Darmstadt, Germany): pancreatin, papain, trichloroacetic acid, sodium acetate, L-cysteine, resazurin, performic acid, 6-aminoquinolyl-N-hydroxysuccinimidyl carbamate, acetonitrile, sodium nitrate, L-tryptophan, hydrochloric acid, ethyl alcohol, and paradimethylaminobenzaldehyde. Buffered peptone water and phosphate-buffered saline (PBS) were obtained from Thermo Fisher Scientific (Waltham, MA, USA), while pepsin enzyme was sourced from Shcharlab S.L. (Barcelona, Spain).

### 2.2. Nutritional Composition of Meat Products 

Cooked meat products used for this study were provided by a local meat industry (ElPozo Alimentación, S.A., Alhama de Murcia, Murcia, Spain). A standard cooked ham and a standard cooked turkey breast already on the market were used as control products. Both of these products were chosen due to their wide popularity in Spain and their good nutritional characteristics within the group of processed meat products. The additives (E-451 triphosphates), E-407 (carrageenans), E-420 (sorbitol), E316 (sodium erythorbate), E-331 (sodium citrate), E-250 (sodium nitrite) and the allergens milk and soya from the standard products were replaced by a 0.3% mix of natural herbal extracts, rich in vitamin C, chlorogenic acids, hydroxytyrosol, catechins, and epicatechins, by 0.3% vinegar and by 3% inulin fibre to obtain the new reformulated products. The nutritional composition of the products is detailed in Table 1.

To produce both standard and reformulated meat products, we followed a conventional production process. Lean pig ham and turkey breast pieces were injected with brine. After injection, they underwent a vacuum tumbling process followed by a 24 h maturation period. Subsequently, a second tumbling process was conducted before stuffing them into 175 mm diameter plastic casings and cooking them for 3 h at temperatures ≥78 °C. For the reformulated products, aimed at achieving necessary extraction and protein stabilization without additives, both vacuum tumbling processes were intensified. This involved increasing the time and tumbling speed by 50% compared to standard processes.

### 2.3. In Vitro Gastrointestinal Digestion

The in vitro digestion method followed was an adaptation of the protocol described by Liu et al. [36], consisting of an in vitro simulation of the gastric and intestinal phase of the human digestion. Firstly, 1 g of meat sample was mixed with 17 mL of 0.1 M HCl and then shredded using a mechanical homogenizer (DI 25 Yellowline, IKA-Werke, Staufen, Germany). To simulate the gastric phase, the pH was adjusted to 2.0, and 1 mL of pepsin solution (2.5 mg/mL in 0.1 M HCl) was added to the mixture and incubated for 2 h in a shaking water bath at 37 °C. For the intestinal phase, the pH was adjusted to 8.0 and then 1 mL of pancreatic solution (2.5 mg/mL in 0.1 M phosphate buffer at pH 8.0) was added, followed by another 2 h incubation in a 37 °C shaking water bath. Once both phases were completed, the tubes were centrifuged (Eppendorf 5804-R Centrifuge, Hamburg, Germany) for 15 min at 9000× *g* at 4 °C to separate supernatant and pellet. Both fractions were stored at −20 °C until use in colonic fermentations.

For the amino acid analysis, the procedure was adapted for 12 g of product. Once digestion was completed, 120 mL of 100 g/L trichloroacetic acid was added to stop gastrointestinal digestion, then the mixture was centrifuged. Finally, pellet and supernatant were stored separately at −80 °C.

### 2.4. In Vitro Gut Microbial Fermentation

The in vitro fermentations were carried out according to the procedure described by Pérez-Burillo et al. [37]. Ten healthy women aged 18–55 years, five of them normal-weight (BMI 18.5–24.9 kg/m^2^) and five obese (BMI ≥ 30 kg/m^2^), donated a faecal sample. Donors who had used antibiotics, pre- or probiotic supplements within the three months prior were not eligible to participate. The faecal samples were pooled together to reduce interindividual variability, and then stored at −20 °C until use.

Faecal slurry from each group was prepared using 16 g of faecal matter homogenised in 70 mL of phosphate buffer (PBS, pH 8.0) previously sterilised. The faecal slurries were homogenised and then centrifuged at 550× *g* for 5 min at 20 °C to take the supernatants for analysis. The solid residue obtained from the in vitro digestion was mixed with 10% of the digestion supernatant to use it as the fermentation substrate. The fermentation medium was prepared consisting of peptone, L-cysteine, and resazurin. Fermentation flasks with 2 mL of faecal slurry, fermentation substrate, and 7.5 mL of fermentation medium, were degassed under an oxygen-free nitrogen stream and incubated in a shaking water bath at 37 °C for 24 h. A baseline sample was collected from the control prior to incubation start and from all other samples and the control after 24 h of incubation. The collected samples were centrifuged at 5000 rpm for 10 min at 4 °C to separate the supernatant and pellet, which were then stored at −80 °C until further use. The resulting solid residues were subsequently used for metagenomic sequencing and the supernatants for short-chain fatty acid analysis.

### 2.5. Amino Acid Analysis

Amino acid analysis of the meat products and the resulting pellet of the in vitro digestion was performed according to the protocol described by Madrid et al. [38]. Samples were lyophilized using a freeze-dryer (LyoMicron, Coolvacum Technologies, Barcelona, Spain). Subsequently, samples were subjected to acid hydrolysis with 6N HCl for 22 h at 112 °C to determine all amino acids except for cysteine, methionine, and tryptophan. Cysteine and methionine were oxidised with performic acid for 16 h at 0 °C and analysed as cysteic acid and methionine sulphone, respectively. Amino acids were separated on a reverse-phase HPLC (Waters 2489 UV/Vis Detector, Waters, Milford, MA, USA). For the determination of primary amino acids, the hydrolysates were derivatised with 6-aminoquinolyl-N-hydroxysuccinimidyl carbamate. The amino acids were separated using a Waters ACCQ.Tag column (3.9 × 150 mm). As for the mobile phases used, solvent A was ACCQ.Tag Eluent A Concentrate Commercial diluted 1:10 in deionised water, and solvent B was a 60% (*v*/*v*) acetonitrile solution. An excitation wavelength of 250 nm and an emission wavelength of 395 nm were set for the fluorescence detector. 

### 2.6. Determination of Tryptophan

The tryptophan content in meat products and the resulting supernatant from in vitro digestion were determined following the protocol described by Morales de León et al. [39]. A mixture of 300 mg of food or 900 mg of digestion supernatant with 12 mL of a 0.4% papain solution in 0.165 M sodium acetate was incubated in a water bath at 65 °C for 16 h. The samples were filtered using Whatman No. 50 filter paper. Then, 4 mL of the filtrate was mixed with 5 mL of a 0.6% paradimethylamino-benzaldehyde solution in 12N HCl. The mixture was left in the dark for 30 min. After that, 5 mL of 96% ethyl alcohol and 5 drops of a 0.2% sodium nitrite solution were added to the tubes. The tubes were left to stand in the dark for an additional 30 min to obtain a blue-violet colour. The absorbance was measured at 620 nm using an ONDA V-11 Scan spectrophotometer (Giorgio Bormac, Carpi, Italy). Calibration curves were obtained by adding tryptophan standards ranging from 0.25 to 4.5 mg of tryptophan per mL, as described above.

### 2.7. In Vitro Digestibility, Digestible Indispensable Amino Acid Ratio (DIAAR), and Digestible Indispensable Amino Acid Score (DIAAS) Calculations

Digestibility represents the proportion of amino acids, measured as a percentage, that are released after the action of gastric and gastrointestinal enzymes. In vitro digestibility for each amino acid were calculated by using the Formula (1):

(1) $$\mathrm{In}\;\mathrm{vitro}\;\mathrm{AA}\;\mathrm{digestibility}\;(\%)=\frac{\left(AA\;in\;food\;\left(mg/100\;g\right)-AA\;in\;food\;pellet\;(mg/100\;g)\right)}{\left(AA\;in\;food\left(mg/100\;g\right)\right)}\times 100$$

In vitro digestibility was calculated for each amino acid (except tryptophan), considering all dilution steps performed during the analysis. The in vitro digestibility of tryptophan was calculated using the Formula (2):

(2) $$\mathrm{Tryptophan}\;\mathrm{in}\;\mathrm{vitro}\;\mathrm{digestibility}\;(\%)=\;\frac{\left(Tryptophan\;in\;food\;supernatant\;(mg/100\;g)\right)}{\left(Tryptophan\;in\;food\;(mg/100\;g)\right)}\times 100$$

From the in vitro AA digestibility, the in vitro DIAAR and DIAAS were calculated. These two indices are the most accurate representation of protein quality and measure the ability of a food to fulfil human amino acid requirements. In vitro DIAAR values were calculated considering the reference protein requirements for older children (from 3 years old), adolescents, and adults given by FAO [25], according to the Formula (3):

(3) $$\mathrm{In}\;\mathrm{vitro}\;\mathrm{DIAAR}\;(\%)=\frac{\left(mg\;of\;IAA\;in\;1\;g\;food\;protein\right)\times in\;vitro\;IAA\;digestibility}{\left(mg\;of\;the\;same\;dietary\;IAA\;in\;1\;g\;of\;the\;reference\;protein\right)}\times 100$$

DIAAR was calculated for all individual indispensable amino acids (IAA). The in vitro DIAAS of a food is the lowest in vitro DIAAR as defined by FAO.

### 2.8. Determination of Short-Chain Fatty Acids 

The production of SCFAs was assessed according to the procedure described in Panzella et al. [40] with some modifications. The supernatants resulting from the in vitro fermentations were analysed by reverse-phase HPLC with diode array detector (1260 Infinity II LC System, Agilent, Santa Clara, CA, USA). The results were obtained in ppm and converted to mmol/kg faeces, expressing the result as the increase in SCFAs in 24 h.

### 2.9. High-Throughput Amplicon Sequencing

Bacterial genomic DNA was isolated from each fermentation pellet sample using NZY Soil gDNA Isolation kit (Nzytech, Lisboa, Portugal). Sequencing of the V3 and V4 regions of the 16S rRNA gene was performed on the MiSeq (Illumina, Essex, UK) platform using 2 × 300 bp reads. The PCR primers targeting the 16S rRNA gene V3 and V4 regions were 5′-TCGTCGGCAGCGTCAGATGTGTATAAGAGACAGCCTACGGGNGGCWGCAG-3′ and 5′-GTCTCGTGGGCTCGGAGATGTGTATAAGAGACAGGACTACHVGGGTATCTAATCC-3′, respectively [41]. The 16S sequencing library was first reviewed with FastQC for overall quality assessment, and the libraries were processed in R package DADA2 [42]. Reads were quality trimmed with the “filterAndTrim” function with “maxEE (2,5)” and reads below 165 bp were discarded. Forward and reverse reads are merged below to generate a table of sequences, and the resulting amplicon sequence variants (ASVs) were subjected to de novo chimera detection using DADA2 and any artifacts were removed.

### 2.10. Bioinformatic Analysis

All obtained amplicon sequence variants (ASVs) were clustered into operate taxonomic units (OTUs) at 97% similarity threshold using QIIME2 v2020.11, and singleton OTUs were discarded [43]. For bacteria taxonomic assignment, centroid ASVs together with their clustered sequences were queried against the Silva database v.132 at 97% similarity using IDTAXA [44] implemented in the R package DECIPHER. The software SPINGO v1.3 was used to assign taxonomy at species level [45]. The abundance matrix, the taxonomy assignment, and the metadata obtained from each sample were merged and imported with the phyloseq v3.12 package [46]. Alpha diversity was calculated in R using the phyloseq package, and several alpha indices were generated, such as OTU numbers, Chao1, ACE, Shannon, Simpson, InvSimpson, and Fisher, and plotted using the function “plot richness”. Beta diversity was calculated using weighted and unweighted UniFrac distances [47]. To test for significant differences in community composition among different seasons, permutational multivariate analysis of variance using distance matrices (PERMANOVA) was conducted using the Adonis function in the R package, and the results were visualised by Principal Coordinates Analysis (PCoA).

### 2.11. Statistical Analysis

The differences between standard and reformulated products were examined using a one-way analysis of variance (ANOVA). Tukey’s test was used to determine significant differences, statistical significance was given at *p* ≤ 0.05 after post hoc comparison. Data processing and statistical analysis were performed using SPSS version 28.0 (IMB Corp., Armonk, NY, USA). For the statistical analysis of the microbial composition, alpha and beta diversity, and SCFAs, the R-studio version 4.2.2 program (Posit PBC, Boston, MA, USA) was used, using the different packages.

## 3. Results and Discussion

### 3.1. Nutritional Composition of Meat Products 

As detailed in Section 2.2, the values for fat, saturated fat, carbohydrates, and salt demonstrate comparable levels between the standard and reformulated products. Nevertheless, the reformulated cooked ham and cooked turkey breast display elevated levels of protein, dietary fibre, antioxidant capacity, and total polyphenols in comparison to their standard counterparts.

### 3.2. Amino Acid Composition of the Products

The amino acid content of each sample can be found in Table 2. The reformulated products exhibited comparable amino acid content per 100 g to their counterparts, with values of 10.14% and 11.24% for ST and SH, respectively. The results of the RT and RH demonstrated an amino acid content of 11.70% and 12.22%, respectively.

Although the reformulated products showed a non-significant increase in their total, indispensable, and branched-chain amino acid content, the proportion of individual amino acids in relation to the total remained unchanged. This consistency is attributed to the fact that the protein matrix of both products is identical, and the additional protein introduced originates from the same meat source. Furthermore, the natural extracts incorporated during reformulation do not contribute exogenous protein. The ratios of essential amino acids in all four products remain consistent, comprising approximately half of the total amino acids. These values slightly exceed those typically reported in the literature, where essential amino acids typically constitute around 42% of total amino acids in pork and turkey [48].

### 3.3. In Vitro Protein Digestibility 

**Figure 1:**
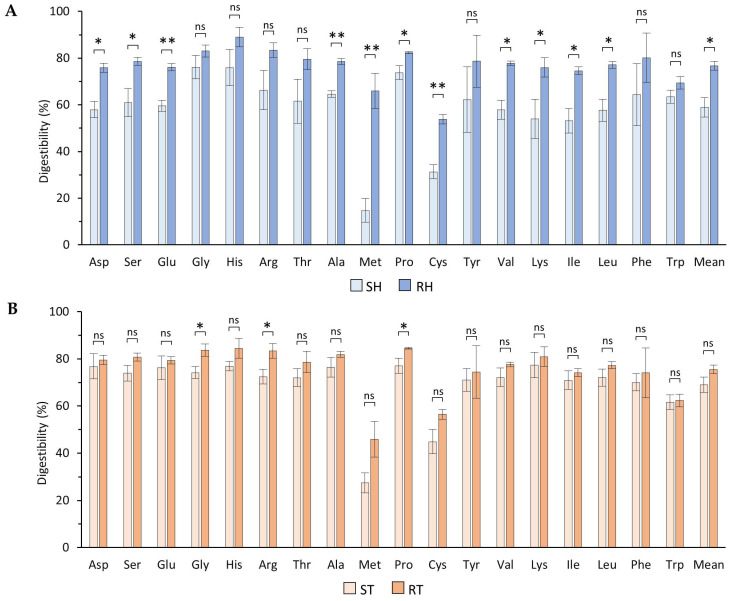
Effect of reformulation on in vitro individual amino acid digestibility. Comparison of the amino acid digestibility of standard and reformulated meat products for cooked turkey breast (**A**) and cooked ham (**B**). The error bars represent the SD of at least two analyses. Significant differences are indicated (ns: non-significant; *: *p* ≤ 0.05; **: *p* ≤ 0.01). Abbreviations of meat products: ST: standard cooked turkey breast, RT: reformulated cooked turkey breast, SH: standard cooked ham, RH: reformulated cooked ham.

The in vitro digestibility of each amino acid was calculated using Formula (1) in Section 2.7. Figure 1 presents the in vitro amino acid digestibility for both standard and reformulated products.

The digestibility of glycine, arginine, and proline in cooked turkey breast significantly increased (*p* ≤ 0.05), as depicted in Figure 1A. No significant trends were observed for the remaining amino acids. In contrast, cooked ham exhibited significant differences in digestibility for aspartic acid, serine, glutamic acid, alanine, methionine, proline, cysteine, valine, lysine, isoleucine, and leucine. Moreover, the average digestibility of amino acids increased significantly from 59% to 77%, as illustrated in Figure 1B. These findings demonstrate that reformulation positively influenced the in vitro digestibility of specific amino acids in the studied meat products. This improvement aligns with previous research; for instance, Faber et al. [49] reported similar total amino acid digestibility rates for chicken breast (83.1%) and pork loin (90.5%).

The natural extracts employed during the reformulation process, including vitamin C, chlorogenic acids, hydroxytyrosol, catechins, and epicatechins, impart the meat products with a high antioxidant capacity [50], exceeding that of the synthetic antioxidants utilised in the standard products, as evidenced in Table 1. Previous studies have indicated that an enhanced antioxidant capacity can facilitate protein digestibility [51,52,53]. Hellwig et al. [54] have proposed that oxygenated free radicals may alter specific aliphatic chains of amino acids, potentially reducing their digestibility during meat storage or processing. Furthermore, oxidation may increase the susceptibility of amino acids such as methionine and cysteine to form disulphide bridges, thereby further reducing their digestibility. This could potentially explain why reformulation has a more pronounced effect on their digestibility, as observed in Figure 1. Moreover, other researchers have employed alternative techniques to enhance the digestibility of protein in meat products. These techniques include various cooking methods [55,56], ultrasound usage [57], fat removal [58], and high-pressure application [59].

### 3.4. In Vitro DIAAR and DIAAS Values

**Figure 2:**
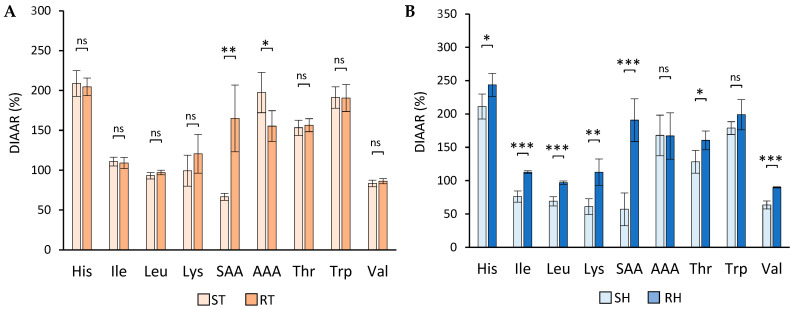
Effect of reformulation on in vitro DIAAR values. Comparison of the DIAAR values of standard and reformulated meat products for cooked turkey breast (**A**) and cooked ham (**B**). SAA: sulphur amino acids (Met + Cys); AAA: aromatic amino acids (Tyr + Phe). The error bars represent the SD of at least two analyses. Significant differences are indicated (ns: non-significant; *: *p* ≤ 0.05; **: *p* ≤ 0.01; ***: *p* ≤ 0.001). Abbreviations of meat products: ST: standard cooked turkey breast, RT: reformulated cooked turkey breast, SH: standard cooked ham, RH: reformulated cooked ham.

The in vitro DIAAR values were calculated using Formula (2) as stated in Section 2.7. Figure 2 presents the digestibility results, comparing the in vitro DIAAR values of the standard and reformulated meat products.

The in vitro DIAAR values for SAA (Met + Cys) substantially increased in cooked turkey breast (Figure 2A). However, no significant increases were observed for any other amino acids, and a decrease for AAA (Tyr + Phe) was noted. In contrast, cooked ham exhibited significant differences in nearly all amino acids (His, Ile, Leu, Lys, SAA, Thr, Trp, and Val) (Figure 2B). The reformulation led to increased DIAAR values, indicating improved satisfaction of certain amino acid requirements in the products. This improvement can be attributed to the higher in vitro digestibility of individual amino acids in cooked ham compared to its standard counterpart. However, this effect was less pronounced for cooked turkey breast, resulting in a smaller impact on DIAAR values. According to FAO guidelines, the DIAAS value of a food is determined by the lowest DIAAR value among all IAAs. Table 3 presents the in vitro DIAAS values for the meat products.

The reformulation significantly improved protein quality for both meat matrices, as indicated by in vitro DIAAS values (*p* ≤ 0.05). Moreover, the reformulation shifted the limiting amino acid from SAA to valine in both meat products. This change primarily stems from the substantial increase in SAA digestibility resulting from the reformulation, as detailed in Figure 1.

However, it is important to recognise that DIAAS values have their limitations. While cooked turkey breast shows notable increases in certain values, there were no significant changes in DIAAR values (as depicted in Figure 2A). This underscores the need for DIAAS values to address malnutrition concerns [60]. Nevertheless, for a comprehensive assessment of protein quality, it is advisable to analyse all DIAAR values collectively.

### 3.5. Gut Microbiota Composition

**Figure 3:**
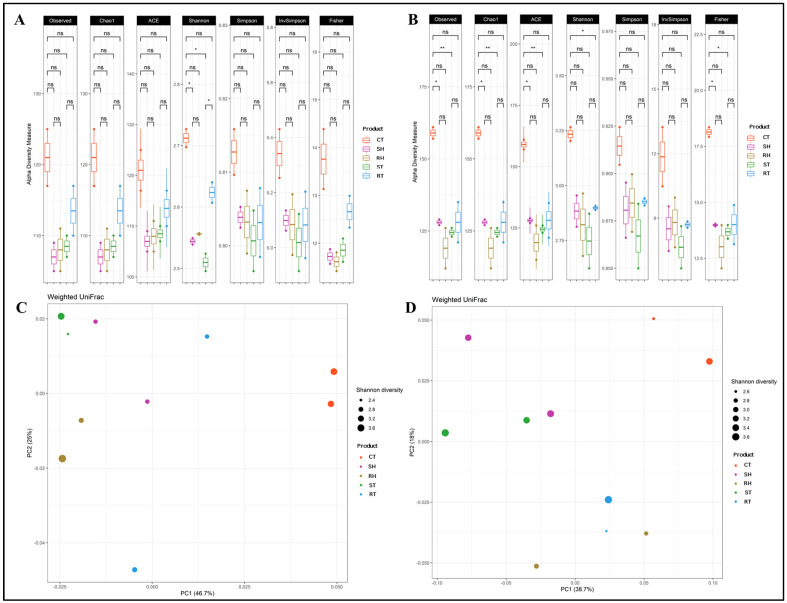
Effect of reformulation in cooked turkey breast and cooked ham on microbial composition. Measurement of alpha diversity of gut microbial communities for normal-weight (**A**) and obese group (**B**). The mean value for each of the following indices is plotted: Observed, Chao1, ACE, Shannon, Simpson, Inverse Simpson, and Fisher. The error bars represent the SD of the two replicates of each sample. Significant differences are indicated (ns: non-significant; *: *p* ≤ 0.05; **: *p* ≤ 0.01). Measurement of beta diversity of gut microbial communities for normal-weight (**C**) and obese group (**D**) using the UniFrac weighted distances (PCoA analysis). Axes represent the two dimensions explaining the greatest proportion of variances in the communities for each analysis. Each point represents the different replicates of the different samples, and their size depends on the diversity value in the Shannon index. Abbreviations of meat products: CT: control, ST: standard cooked turkey breast, RT: reformulated cooked turkey breast, SH: standard cooked ham, RH: reformulated cooked ham.

Following in vitro fermentation of digested cooked ham and cooked turkey breast samples, the microbial community structure was analysed for both the normal-weight and obese groups. The relative abundance of bacterial communities, as well as alpha and beta diversity were evaluated. The results for the microbial community composition are detailed in Figure 3.

#### 3.5.1. Alpha and Beta Diversity

In terms of alpha diversity within the bacterial community, the control group exhibited higher diversity measures compared to the meat products for the normal-weight group (see Figure 3A). However, both reformulated meat products showed higher diversity values across all indices compared to their standard counterparts, with statistical significance observed only for reformulated cooked turkey breast in the Shannon index. For the obese group (refer to Figure 3B), similar trends were observed where the control group displayed higher diversity than the meat products. Comparing reformulated and standard meat products, reformulated cooked turkey breast showed lower diversity measures across most indices, whereas reformulated cooked ham exhibited higher diversity in all indices. Previous studies have indicated that meat or meat products may adversely affect gut microbiota diversity due to certain properties of animal protein [61].

Furthermore, beta diversity among inter-community bacteria was assessed using weighted UniFrac distances in PCoA ordination analysis. This method measures differences between samples based on the distances between them, indicating closer similarity between more similar samples. Figure 3C illustrates that microbial community structures of the meat products in the normal-weight group were similar, while distinct from the control group. The reformulated products showed some divergence from their standard counterparts. Similarly, Figure 3D for the obese group reflected outcomes similar to the normal-weight group, highlighting discernible discrimination between standard and reformulated products in this context.

#### 3.5.2. Relative Abundance

**Figure 4:**
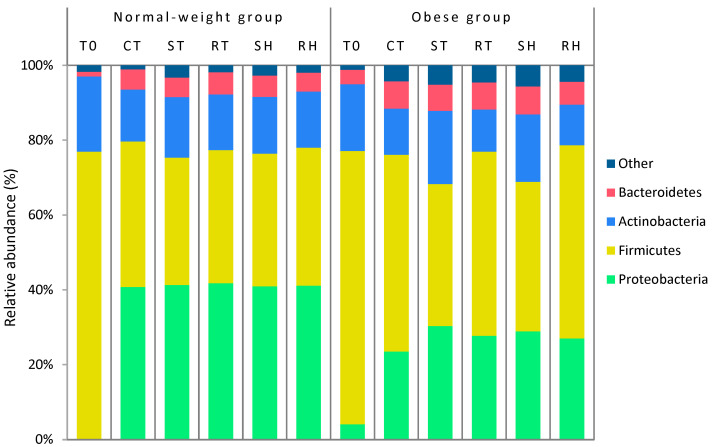
Bar graph of gut microbial community structure at phylo level in percentage of relative abundance in both normal-weight and obese group. Abbreviations of meat products: CT: control, ST: standard cooked turkey breast, RT: reformulated cooked turkey breast, SH: standard cooked ham, RH: reformulated cooked ham.

Figure 4 depicts the bacterial composition at the phylogenetic level, while Table 4 outlines significant differences in relative abundance observed at the phylogenetic, genus, and OTU levels between standard and reformulated products for both participant groups.

In the normal-weight group, no statistically significant differences were observed in any matrices at the phylogenetic level due to reformulation. However, at the genus level, there was a notable increase (*p* < 0.05) in *Collinsella* in both meat products as a result of reformulation. Specifically, one OTU (OTU_123), identified as *Collinsella aerofaciens*, showed a significant increase in both reformulated products at the OTU level. *Collinsella* genera have historically been associated with various inflammatory disorders [62], but recent studies indicate that *Collinsella aerofaciens* can ferment carbohydrates in the colon, producing ethanol, short-chain fatty acids, and lactate [63,64].

On the opposite, the group with obesity exhibited more pronounced differences. Reformulation led to a significant decrease in *Actinobacteria* and an increase in *Firmicutes* at the phylogenetic level for both meat products. At the genus level, *Bifidobacterium* showed a significant decrease in both reformulated products. At the OTU level, the only significant decrease observed due to reformulation in both meat products was OTU_38 (*Bifidobacterium adolescentis*). *Bifidobacterium adolescentis* is a common probiotic species in the adult human gut [65,66], and its reduction may be attributed to the variable effects of additives on different intestinal bacteria.

In the normal-weight group, the study found that reformulations resulted in minor changes in the composition and diversity of the bacterial communities. These changes seem to be associated with the replacement of certain additives that may affect the balance of the gut microbiota, the mucous layer, or both [67,68,69]. E-407 (carrageenan) is a controversial additive that has been linked to intestinal issues, decreased production of short-chain fatty acids, and modifications to the mucous layer [11,70]. To reduce the negative impact of conventional products on gut health, it may be beneficial to substitute additives and allergens with natural extracts such as dietary fibre and antioxidants. It is important to note that all evaluations are objective and free from bias.

### 3.6. Production of Short-Chain Fatty Acids (SCFAs) 

SCFAs were analysed for two participant groups after 24 h of in vitro fermentation of samples of cooked turkey breast and cooked ham, as well as a water blank. Table 5 shows the production of acetic acid, propionic acid, butyric acid, and total SCFAs.

Reformulation resulted in significant increases (*p* ≤ 0.05) in the production of acetic acid, propionic acid, and total short-chain fatty acids (SCFA) for cooked turkey breast in the normal-weight group. Cooked ham also exhibited a significant increase in propionic acid and total SCFA production. However, there were no significant differences observed in butyric acid production for either product. In contrast, the obese group showed a significant increase only in acetic acid production for cooked turkey breast after reformulation, with no significant changes in SCFAs for cooked ham. Overall, the normal-weight group experienced greater increases in total SCFA production compared to the obese group.

These changes can be linked to specific bacterial genera. Previous studies have established associations between *Bifidobacterium* and *Escherichia-Shigella* genera with acetic acid synthesis, while *Bacteroides* and *Paraclostridium* are associated with propionic and butyric acid production, respectively [71,72,73]. The absence of carrageenan in reformulated products could potentially influence changes in the abundance of these bacterial groups. The study suggests that the beneficial effects of natural extracts were less pronounced in the obese group compared to the normal-weight group, possibly due to the influence of body weight on gut microbiota, known as dysbiosis in obesity [74].

However, substituting additives with natural extracts did not induce as many changes in microbial composition as anticipated. This may be attributed to the high protein content and digestibility of the new products. Research indicates that increased protein and nitrogen intake could potentially adversely affect gut health [75]. Additionally, limitations of in vitro analyses should be considered, as the intake of a single food may not fully represent the overall dietary context.

## 4. Conclusions

The study found that using natural ingredients and removing additives and allergens improved digestibility and protein quality. Specifically, in cooked ham, the removal of additives improved protein quality in 7 out of 9 IAAs, indicating better-met requirements. While there may be concerns about the impact of increased protein digestibility on gut microbiota, our study found no changes in microbial composition. The study found that the production of specific genera and short-chain fatty acids (SCFAs) improved. It is worth noting that the use of additives in regulated amounts should not cause undue concern among consumers. The results indicate that replacing additives with natural extracts could have a positive impact on human health. Due to the results obtained, it can be considered that the absence of additives in this type of product may have long-term effects on the intestinal and even general health of consumers. For this reason, further research on the possible effects of free additives in meat products is necessary in humans through in vivo studies.

## Figures and Tables

**Table 1:** Nutritional composition of meat products.

	ST	RT	SH	RH
Energy value (KJ/Kcal/100 g)	292/69	379/89	387/92	405/95.7
Fats (%)	1	0.7	1.5	1.5
Saturated fats (%)	0.3	0.2	0.5	0.5
Carbohydrates (%)	0.5	0.5	1.5	1.0
Sugars (%)	0.5	0.3	1.3	0.4
Proteins (%)	14.5	20.2	18.0	20.0
Salt (%)	1.8	1.6	1.9	1.7
Dietary fibre (%)	0	0.3	0	0.3
Antioxidant capacity (FRAP (µM Trolox/100 g))	1199	1700	1019	1741
Total polyphenols (mg gallic acid/100 g)	172	403	223	342

Abbreviations of meat products: ST: standard cooked turkey breast, RT: reformulated cooked turkey breast, SH: standard cooked ham, RH: reformulated cooked ham.

**Table 2:** Amino acid composition of meat products (g/100 g of meat product).

	ST	RT	SH	RH
**Dispensable AA**				
Asp	0.83 ± 0.14	0.88 ± 0.21	0.79 ± 0.12	0.98 ± 0.18
Ser	0.49 ± 0.01	0.51 ± 0.00	0.51 ± 0.04	0.54 ± 0.01
Glu	1.33 ± 0.19	1.42 ± 0.29	1.32 ± 0.20	1.58 ± 0.23
Gly	0.53 ± 0.02	0.66 ± 0.02	0.92 ± 0.05	0.70 ± 0.07
Arg	0.84 ± 0.04	0.98 ± 0.02	0.97 ± 0.02	1.02 ± 0.05
Ala	0.50 ± 0.03	0.61 ± 0.03	0.60 ± 0.06	0.66 ± 0.10
Pro	0.42 ± 0.03	0.46 ± 0.02	0.62 ± 0.03	0.52 ± 0.07
**Total ^1^**	4.93 ± 0.34	5.51 ± 0.52	5.73 ± 0.58	6.01 ± 0.24
	(49%)	(49%)	(49%)	(49%)
**Indispensable AA**				
His	0.44 ± 0.05	0.44 ± 0.09	0.52 ± 0.07	0.54 ± 0.07
Thr	0.54 ± 0.03	0.56 ± 0.21	0.61 ± 0.36	0.62 ± 0.14
Lys	0.63 ± 0.01	0.80 ± 0.01	0.63 ± 0.04	0.87 ± 0.04
Phe	0.65 ± 0.01	0.54 ± 0.06	0.71 ± 0.10	0.59 ± 0.04
Tyr	0.51 ± 0.08	0.42 ± 0.02	0.57 ± 0.03	0.46 ± 0.14
Met	0.35 ± 0.01	0.67 ± 0.02	0.64 ± 0.05	0.64 ± 0.00
Cys	0.13 ± 0.14	0.21 ± 0.19	0.20 ± 0.11	0.20 ± 0.19
Trp	0.21 ± 0.01	0.23 ± 0.04	0.22 ± 0.05	0.23 ± 0.01
Ile	0.48 ± 0.01	0.50 ± 0.03	0.50 ± 0.08	0.55 ± 0.03
Leu	0.80 ± 0.09	0.86 ± 0.03	0.86 ± 0.04	0.94 ± 0.13
Val	0.47 ± 0.01	0.50 ± 0.02	0.51 ± 0.01	0.57 ± 0.02
**Total IAA ^2^**	5.20 ± 0.11	5.73 ± 0.09	5.97 ± 0.84	6.21 ± 0.07
	(51%)	(51%)	(51%)	(51%)
**Total BCAA ^3^**	1.74 ± 0.01	1.86 ± 0.09	1.87 ± 0.17	2.06 ± 0.03
	(17%)	(17%)	(16%)	(17%)
**Total AA ^4^**	10.14 ± 0.23	11.24 ± 0.43	11.70 ± 1.43	12.22 ± 0.17

^1^ Total: combined total of dispensable amino acids, including Asp, Ser, Glu, Gly, Arg, Ala, and Pro. ^2^ Total IAA: combined total of indispensable amino acids, including His, Thr, Lys, Phe, Tyr, Met, Cys, Trp, Ile, Leu, and Val. ^3^ Total BCAA: combined total of branched-chain amino acids (Ile, Leu, and Val). ^4^ Total AA: combined total of amino acids. The results are shown as mean ± SD of duplicate determinations. **Abbreviations of meat products**: ST: standard cooked turkey breast, RT: reformulated cooked turkey breast, SH: standard cooked ham, RH: reformulated cooked ham. **Abbreviations of amino acids**: Asp: asparagine, Ser: serine, Glu: glutamine, Gly: glycine, Arg: arginine, Ala: alanine, Pro: proline, His: histidine, Thr: threonine, Lys: lysine, Phe: phenylalanine, Tyr: tyrosine, Met: methionine, Cys: cysteine, Trp: Tryptophan, Ile: isoleucine, Leu: leucine, Val: valine.

**Table 3:** Effect of reformulation on in vitro DIAAS values.

Meat Product	DIAAS (%)	Limiting IAA ^1^	*p*-Value ^2^
ST	66.68 ± 4.39	SAA (Met + Cys)	0.001
RT	86.24 ± 3.24	Val	
SH	57.14 ± 24.59	SAA (Met + Cys)	0.037
RH	90.05 ± 0.91	Val	

^1^ Limiting IAA is the indispensable amino acid that presents the lowest DIAAR value. ^2^ *p*-values between standard and reformulated products were examined using a one-way analysis of variance (Tukey’s test), *p*-values < 0.05 indicate that significant differences exist due to reformulation. The results are shown as mean ± SD of duplicate determinations. **Abbreviations of meat products**: ST: standard cooked turkey breast, RT: reformulated cooked turkey breast, SH: standard cooked ham, RH: reformulated cooked ham.

**Table 4:** Impacted microbial features at phylo, genus, and OTU levels.

		Blank	Cooked Turkey Breast			Cooked Ham		
			Control	Reformulated	*p*-Value ^1^	Control	Reformulated	*p*-Value ^1^
**Normal-Weight Group**								
**Genus**								
*Collinsella*		1.62 ± 0.24	1.6 ± 0.08	2.56 ± 0.23	**0.019**	1.77 ± 0.09	2.88 ± 0.23	**0.011**
**OTU**								
Otu_73	*Dorea longicatena*	0.08 ± 0.07	0.10 ± 0.08	0.30 ± 0.03		0.08 ± 0.03	0.44 ± 0.03	**0.006**
Otu_123	*Collinsella aerofaciens*	0.33 ± 0.02	0.03 ± 0.00	0.62 ± 0.03	**0.001**	0.04 ± 0.00	1.07 ± 0.07	**0.002**
Otu_173	*Blautia obeum*	0.83 ± 0.06	0.66 ± 0.04	0.49 ± 0.01	**0.022**	0.62 ± 0.27	0.70 ± 0.15	
Otu_228	*Butyricimonas paravirosa*	0.14 ± 0.01	0.12 ± 0.01	0.19 ± 0.02	**0.031**	0.18 ± 0.03	0.13 ± 0.03	
**Obese group**								
**Phylo**								
Firmicutes		52.55 ± 5.25	37.93 ± 2.76	49.20 ± 0.47	**0.030**	39.94 ± 5.69	51.67 ± 1.04	
Actinobacteria		12.33 ± 0.33	19.60 ± 0.44	11.24 ± 0.04	**0.001**	18.04 ± 0.20	10.81 ± 1.58	**0.023**
**Genus**								
*Adlercreutzia*		1.00 ± 0.14	0.61 ± 0.04	1.30 ± 0.12	**0.014**	0.82 ± 0.08	0.97 ± 0.19	
*Bifidobacterium*		10.24 ± 0.02	18.02 ± 0.28	8.66 ± 0.20	**<0.001**	15.98 ±0.35	8.08 ± 0.53	**<0.001**
*Parabacteroides*		3.52 ± 0.20	2.38 ± 0.22	1.87 ± 0.11		2.50 ± 0.35	1.43 ± 0.09	**0.021**
**OTU**								
Otu_38	*Bifidobacterium adolescentis*	4.02 ± 6.33	12.40 ± 0.26	3.20 ± 0.11	**<0.001**	9.76 ± 0.09	2.80 ± 0.23	**0.001**
Otu_74	*Adlercreutzia equolifaciens*	1.00 ± 0.33	0.61 ± 0.04	1.30 ± 0.12	**0.017**	0.82 ± 0.08	0.97 ± 0.19	
Otu_83	*Bifidobacterium bifidum*	0.14 ± 0.04	0.08 ± 0.01	0.08 ± 0.01		0.10 ± 0.00	0.09 ± 0.00	**0.029**
Otu_140	*Bacteroides eggerthii*	0.38 ± 0.06	0.41 ± 0.07	0.30 ± 0.05		0.49 ± 0.05	0.29 ± 0.03	**0.040**
Otu_154	*Eggerthella lenta*	0.04 ± 0.10	0.18 ± 0.00	0.27 ± 0.02	**0.031**	0.25 ± 0.02	0.25 ± 0.01	
Otu_183	*Bacteroides nordii*	0.17 ± 0.06	0.12 ± 0.01	0.22 ± 0.00	**0.003**	0.15 ± 0.02	0.15 ± 0.02	
Otu_228	*Butyricimonas paravirosa*	0.49 ± 0.01	0.44 ± 0.13	0.50 ± 0.01		0.68 ± 0.04	0.52 ± 0.00	**0.037**

^1^ *p*-values between standard and reformulated products were examined using a one-way analysis of variance (Tukey’s test). Significant *p*-values (*p* < 0.05) are presented in bold. Significant *p*-values indicate the differences of the bacteria production due to reformulation. The results are shown as mean percentage of relative abundance ± SD of duplicate determinations.

**Table 5:** Effect of reformulation on SCFA production (mM).

	Acetic Acid	Propionic Acid	Butyric Acid	Total SCFA
**Normal-weight group**				
Control	20.60 ± 0.78 ^b^	10.38 ± 0.85 ^b^	4.10 ± 1.64 ^a^	35.08 ± 1.71 ^b^
ST	24.65 ± 0.82 ^b^	12.07 ± 0.76 ^ab^	3.92 ± 0.22 ^a^	40.63 ± 1.81 ^b^
RT	34.13 ± 5.62 ^a^	14.80 ± 0.28 ^a^	4.10 ± 0.46 ^a^	53.03 ± 6.36 ^a^
SH	25.71 ± 4.53 ^ab^	5.72 ± 2.00 ^c^	3.60 ± 0.40 ^a^	35.03 ± 6.94 ^b^
RH	26.46 ± 1.03 ^ab^	12.32 ± 1.54 ^ab^	4.13 ± 0.46 ^a^	42.90 ± 3.03 ^ab^
**Obese group**				
Control	18.66 ± 1.49 ^c^	7.38 ± 5.78 ^a^	2.79 ± 1.02 ^a^	28.83 ± 8.29 ^a^
ST	21.30 ± 0.43 ^c^	3.46 ± 4.89 ^a^	4.71 ± 2.07 ^a^	29.47 ± 3.25 ^a^
RT	31.65 ± 2.16 ^a^	1.31 ± 1.85 ^a^	3.17 ± 0.60 ^a^	36.13 ± 4.61 ^a^
SH	25.41 ± 1.36 ^b^	1.67 ± 1.14 ^a^	2.30 ± 0.76 ^a^	29.39 ± 1.75 ^a^
RH	27.84 ± 0.57 ^b^	2.49 ± 0.36 ^a^	3.78 ± 2.54 ^a^	34.11 ± 1.61 ^a^

The results are shown as mean of duplicate determinations ± SD. ^a–c^ Different letters in superscript within the same row indicate statistically significant differences (*p* < 0.05). Abbreviations of meat products: CT: control, ST: standard cooked turkey breast, RT: reformulated cooked turkey breast, SH: standard cooked ham, RH: reformulated cooked ham.

## Data Availability

The original contributions presented in the study are included in the article, further inquiries can be directed to the corresponding author.
